# Quantitative Myocardial Perfusion with Dynamic Contrast-Enhanced Imaging in MRI and CT: Theoretical Models and Current Implementation

**DOI:** 10.1155/2016/1734190

**Published:** 2016-03-10

**Authors:** G. J. Pelgrim, A. Handayani, H. Dijkstra, N. H. J. Prakken, R. H. J. A. Slart, M. Oudkerk, P. M. A. Van Ooijen, R. Vliegenthart, P. E. Sijens

**Affiliations:** ^1^University Medical Center Groningen, Center for Medical Imaging North-East Netherlands (CMI-NEN), University of Groningen, Hanzeplein 1, 9713 GZ Groningen, Netherlands; ^2^University Medical Center Groningen, Department of Radiology, University of Groningen, Hanzeplein 1, 9713 GZ Groningen, Netherlands; ^3^University Medical Center Groningen, Department of Nuclear Medicine and Molecular Imaging, University of Groningen, Hanzeplein 1, 9713 GZ Groningen, Netherlands

## Abstract

Technological advances in magnetic resonance imaging (MRI) and computed tomography (CT), including higher spatial and temporal resolution, have made the prospect of performing absolute myocardial perfusion quantification possible, previously only achievable with positron emission tomography (PET). This could facilitate integration of myocardial perfusion biomarkers into the current workup for coronary artery disease (CAD), as MRI and CT systems are more widely available than PET scanners. Cardiac PET scanning remains expensive and is restricted by the requirement of a nearby cyclotron. Clinical evidence is needed to demonstrate that MRI and CT have similar accuracy for myocardial perfusion quantification as PET. However, lack of standardization of acquisition protocols and tracer kinetic model selection complicates comparison between different studies and modalities. The aim of this overview is to provide insight into the different tracer kinetic models for quantitative myocardial perfusion analysis and to address typical implementation issues in MRI and CT. We compare different models based on their theoretical derivations and present the respective consequences for MRI and CT acquisition parameters, highlighting the interplay between tracer kinetic modeling and acquisition settings.

## 1. Introduction

Myocardial perfusion imaging (MPI) is commonly used to investigate myocardial ischemia. While different modalities for MPI have different diagnostic accuracy, the overall accuracy to diagnose hemodynamically significant coronary artery disease (CAD) is good [1]. Analysis of MPI results in the clinical setting is mostly performed by visual evaluation of presence and pattern of hypoenhancement of the myocardium during first-pass of intravenously injected contrast. Presence of regions with normal perfusion is essential for this method to work. This is a limitation for diagnosis of patients with multivessel disease or balanced ischemia [2]. MPI can only distinguish multivessel disease and balanced ischemia when quantitative measures of myocardial perfusion are provided.

Positron emission tomography (PET) was the first technique to establish quantitative measures for perfusion. In PET, time-resolved acquisition of the first-pass of tracer uptake and direct quantification of tracer concentration were developed. With those parameters quantified, tracer kinetic modeling (1-compartment or 2-compartment modeling) could be applied to produce independent estimates of perfusion in stress and rest, known as absolute perfusion measurement (mL/g/min). This technique has been validated using microsphere comparison [2–4]. Furthermore, added clinical value beyond relative and visual perfusion analysis has been demonstrated [5–8]. The myocardial perfusion reserve (MPR), calculated from PET-derived perfusion measurement at stress and rest, was shown to be an important predictor of cardiovascular events [9–11].

A limitation of cardiac PET is the relatively high cost and the need for an on-site cyclotron, depending on the tracer. Recent developments with the new ^18^F-tracer flurpiridaz or other improved tracers could obviate the need for an on-site cyclotron. Flurpiridaz has shown good linearity of myocardial uptake with perfusion at a large flow range, excellent myocardial retention, low background noise in adjacent organs, and a relatively long half-life (110 min) [12].

Magnetic resonance imaging (MRI) and computed tomography (CT) imaging could be important modalities to compete with PET for the complete workup of cardiac patients. Recent developments have sparked interest for myocardial perfusion quantification using these techniques. State-of-the-art MRI and CT have better spatial and temporal resolution compared to PET. Integration of MRI and CT into current workup for coronary artery disease (CAD) also profits from their wider availability, lower costs, and increasing clinical role in comprehensive diagnosis of CAD. The validity and noninferiority of MRI and CT compared to PET measurements need to be demonstrated before a decision regarding the preference for MRI or CT over PET for myocardial perfusion quantification can be reached. Lack of standardized acquisition and modeling protocols for myocardial perfusion acquisition have complicated comparison between studies and modalities.

The aim of this study is to provide insight into the tracer kinetic models in absolute myocardial perfusion quantification, and their implementation requirements for CT and MRI. A further aim was to analyze the factors that influence myocardial perfusion quantification.

## 2. Myocardial Perfusion Imaging in MRI and CT

Perfusion refers to the delivery of blood to the tissue via the intravascular capillary pathway. Perfusion imaging uses dynamic contrast-enhanced acquisition to observe the first-pass dynamics of contrast agent delivery into the tissue of interest over time. For myocardial perfusion quantification, the first-pass contrast dynamics at the respective supplying artery or other arterial input sites should be captured as well. The typical arterial input sites for myocardial perfusion are the left ventricular cavity in MRI or the descending aorta in CT.

### 2.1. Contrast Agent

MRI and CT use different agents (gadolinium and nonionic iodine, resp.) to acquire contrast in the myocardial perfusion scans: both small molecules (<1 kDa, typical particle diameters of 0.82 nm for gadolinium dimeglumine and 1.4 nm of iohexol) that distribute to the interstitial space and generally do not enter the intracellular space. Actually, MRI contrast agents do interact with the intracellular space by changing the relaxivity of water that diffuses freely into the cell. The diffusion constants for MRI and CT contrast agents are roughly similar: 2.7 × 10^−2^ m^2^/s for gadolinium dimeglumine, and 2.5 × 10^-2 ^m^2^/s for iohexol [13, 14]. Although the nonionic iodine contrast agents typically have a much higher viscosity compared to gadolinium, neither CT nor MRI contrast agents have significant effects on the viscosity of the blood stream [15, 16]. Gadolinium-based contrast agents have limited linearity of contrast enhancement to contrast concentration, with higher dose resulting in blood signal saturation [17, 18]. A typical dose of gadolinium contrast for visual and quantitative analysis is 0.05 mmol/kg at an injection rate of 4-5 mL/s. Dosages as low as 0.03 mmol/kg body weight have been recommended to prevent contrast saturation both in the myocardium and in the arterial input function [19]. Iodine-based contrast agents on the other hand have more straightforward and steady linearity of contrast enhancement to contrast concentration, which greatly simplifies absolute quantification [20]. In myocardial perfusion studies with multidetector CT, iodine contrast agents are administered at an injection rate of 3–5 mL/s and a volume of 60–70 mL [21, 22]. The resulting lengthy administration of CT contrast (longer than the first-pass of diffusion), however, violates the principles of indicator dilutor theory and will affect the accuracy of quantification.

### 2.2. Acquisition of Myocardial Perfusion Imaging

In MRI, myocardial perfusion imaging is mainly based on T1-weighted pulse sequences, where interactions of paramagnetic gadolinium (Gd^3+^) with surrounding water molecules result in lower T1 relaxation times of the protons involved, resulting in signal enhancement showing as hyperintensity on the T1-weighted image, reflecting the distribution of gadolinium [23]. The current protocol allows acquisition of three myocardial short-axis slices at every heartbeat with a typical spatial resolution of 1.5 × 1.5 × 10 mm^3^, performed during 50–60 consecutive heartbeats. In dynamic contrast-enhanced MRI, temporal resolution generally is in the order of 1 sec (1-2 cardiac cycles). Recently introduced advanced accelerated imaging sequences achieve whole heart 3D perfusion MRI with a voxel resolution of 2.3 × 2.3 × 5 mm^3^, although with their reduced temporal resolution not a substitute for quantitative estimation of myocardial blood flow [24]. In CT, with dynamic shuttling mode acquisition, whole heart coverage with higher spatial resolution can be obtained (0.3 × 0.3 × 5 mm^3^) at every 2-3 heartbeats [22]. In contrast, CT scanners with wider detectors, of up to 16 cm, can achieve whole heart acquisition in a single heartbeat [25]. In both methods, perfusion imaging is performed over 20–30 consecutive heartbeats. CT has a high temporal resolution. The latest generation of dual-source CT scanners has a temporal resolution per acquisition of approximately 63 ms. However, dual-source CT scanners need to shuttle between two positions, resulting in a time interval in-between scans of once every second heartbeat and, for high heart rhythms, once every three cardiac cycles. The 256- and 320-slice CT scanners have lower temporal resolution per acquisition (in the order of 135 ms), but these scanners do not have to shuttle between two positions in order to acquire information about the whole heart, providing the opportunity to image at every heartbeat (at the cost of higher dose). A limitation in CT, and especially also in dynamic CT perfusion studies, is that the radiation dose is directly related to the number of images acquired. For a thorough overview of CT perfusion acquisition techniques one is referred to the review by Rossi et al. [26].

### 2.3. Why Use Modeling in MRI and CT

In theory, tissue perfusion can be inferred from the apparent contrast enhancement without any complex modeling, assuming that the contrast agent is hemodynamically inert. This hypothesis holds true if two criteria are met with (1) a linear relationship between contrast enhancement and contrast concentration (*ex vivo* linearity) and (2) a linear relationship between apparent contrast enhancement and* perfusion* (*in vivo/uptake* linearity).

*Ex vivo* linearity is present in PET tracers as well as in CT iodine-based contrast agents regardless of their concentrations and in MR gadolinium only up to a certain concentration limit [17, 18].* In vivo/uptake* linearity is limited in case of extravasating contrast agent. Most contrast agents in perfusion imaging do not only flow to the intravascular space but also distribute to the extracellular extravascular space (EES). Only in case EES extraction fraction is constant within the range of physiological perfusion flow, the apparent contrast enhancement will be linear to perfusion, as is the case with ^15^O-water and ^18^F-flurpiridaz in PET. However, in most tracers such as ^13^N-ammonia [12, 23, 27], ^82^Rb-rubidium [12, 23], gadolinium [23, 28–30], and iodine [23, 31, 32], the extraction fractions decrease nonlinearly with increasing perfusion, causing reduced* in vivo/uptake* linearity.

To correct for the effect of these extravasating tracers or contrast agents on contrast enhancement, tracer kinetic modeling attempts to separate the dynamics of contrast agent in the intravascular space and the EES over time to yield more accurate perfusion estimation. These modeling techniques have been successfully applied in PET myocardial perfusion imaging with different tracers, including ^13^N-ammonia and ^82^Rb-rubidium [33]. It is theoretically feasible to implement the same principles in MRI and CT, using tracer kinetic modeling.

## 3. Tracer Kinetic Modeling

**Figure 1:**
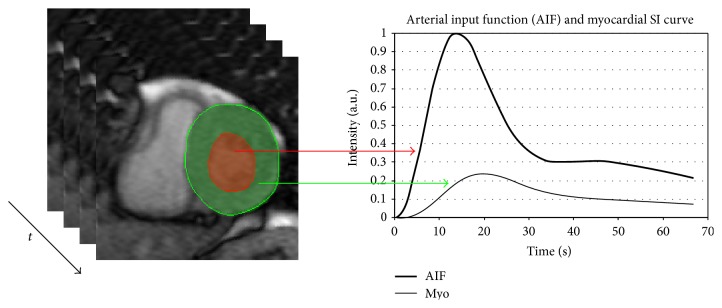
Myocardial (green voxels) and arterial input function (red voxels) tracing to produce contrast dynamics time curves.

Tracer kinetic modeling essentially relates the dynamics of tracer or contrast agent concentration in tissue (myocardium) to that in the supplying artery referred to as arterial input function (AIF). The contrast dynamics over time are obtained by tracing the myocardium and AIF voxels from the dynamic contrast-enhanced acquisition (Figure 1).

**Figure 2:**
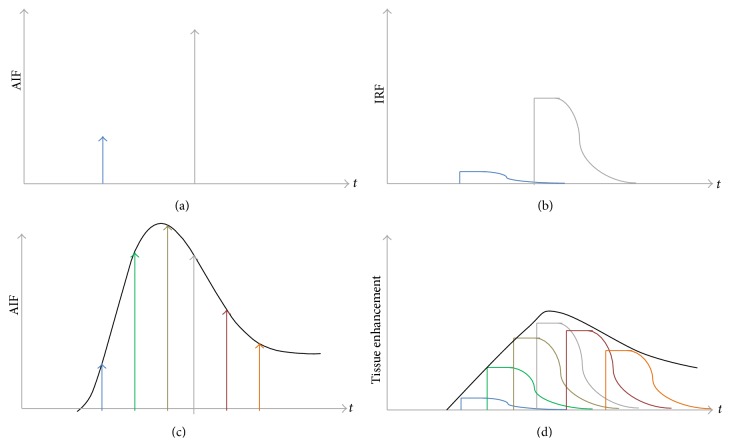
Single arterial inlets are shown with different magnitude scale in different time instance (a) and the respective magnitude-scaled impulse response function (IRF) in the tissue (b). A contrast bolus can be modeled as trains of arterial inlets (c), producing trains of magnitude-scaled IRF in the tissue (d). Deconvolution aims to reconstruct the IRF that fits the relation between the red and green lines in (c) and (d), respectively.

The mathematical relation between contrast dynamics in the tissue and in the AIF is represented by an impulse response function (IRF) (Figure 2). As a result of a one unit-amplitude of an infinitely narrow input bolus (impulse bolus) in the arterial inlet (Figure 2(a)), contrast retention will occur in the tissue with a certain dynamic proportion in time, defined as IRF (Figure 2(b)). In a perfusion imaging study, the AIF can be considered as a train of time-shifted and magnitude-scaled impulse boluses (Figure 2(c)) producing a corresponding train of time-shifted and magnitude-scaled IRFs in the tissue (Figure 2(d)). An iterative curve-fitting operation called deconvolution can then be applied to reconstruct the IRF from the AIF and tissue enhancement curves. Since deconvolution may lead to more than one mathematically suitable solution, it is necessary to restrict the operation by requiring the IRF to follow a certain parameterized formulation specific for each perfusion model. Therefore, perfusion flow is estimated from those IRF parameters providing the best fit at deconvolution.

## 4. Different Tracer Kinetic Models

Tracer kinetic models for absolute myocardial perfusion quantification can be classified into three model groups: distributed parameter, compartmental, and indicator dilution theory approaches, each of which has been developed into more specific models. For thorough explanation of the distributed parameter and compartmental models one is referred to the technical paper by Sourbron and Buckley and two manuscripts by Jerosch-Herold for the indicator dilution theory approach [34–36]. In the present overview those modeling approaches are solely compared on the basis of physical interpretation of their respective IRF.

**Figure 3:**
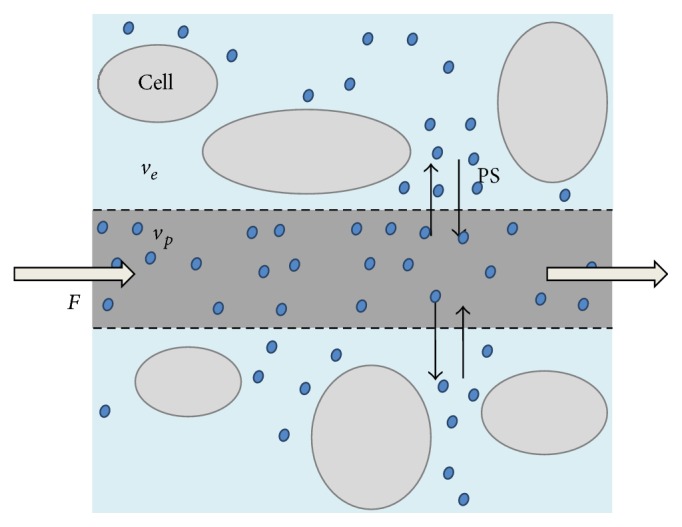
Illustration of contrast agent (blue dots) distribution in the tissue: *v*_*p*_ is the plasma volume within the intravascular space, *v*_*e*_ is the extravascular extracellular space, *F* is the perfusion flow within the intravascular space, and PS is the permeability-surface exchange rate between *v*_*p*_ and *v*_*e*_. Another parameter, the extraction fraction (*E*), denotes the proportion of contrast agent exchanged to the extravascular extracellular space.

**Figure 4:**
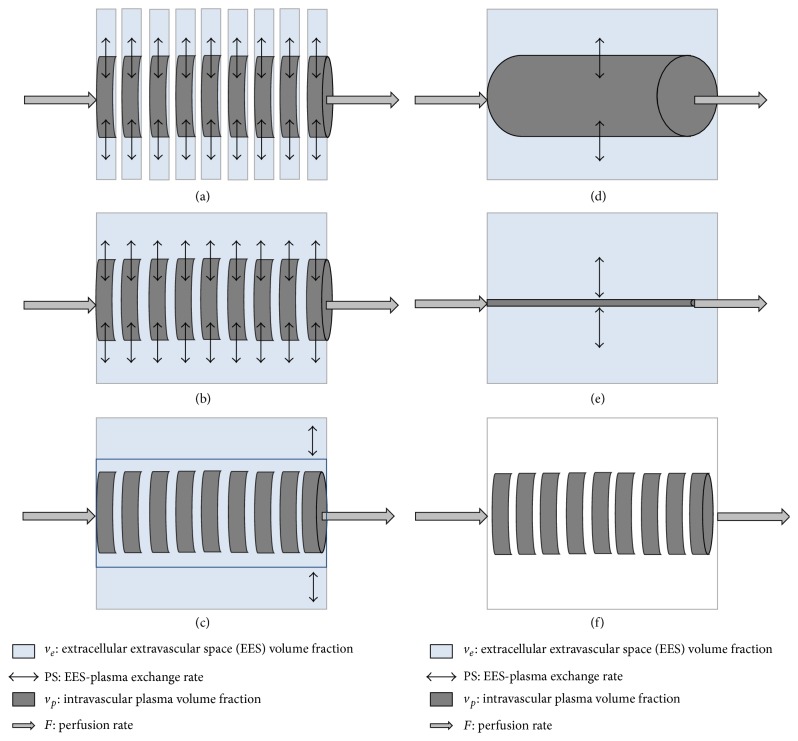
Schematic representation of different tracer kinetic models: (a) distributed parameter model, (b) tissue homogeneity model, (c) adiabatic approximation of tissue homogeneity model, (d) 2-compartment model, (e) 1-compartment (Toft's) model, and (f) Fermi model.

**Figure 5 fig5:**
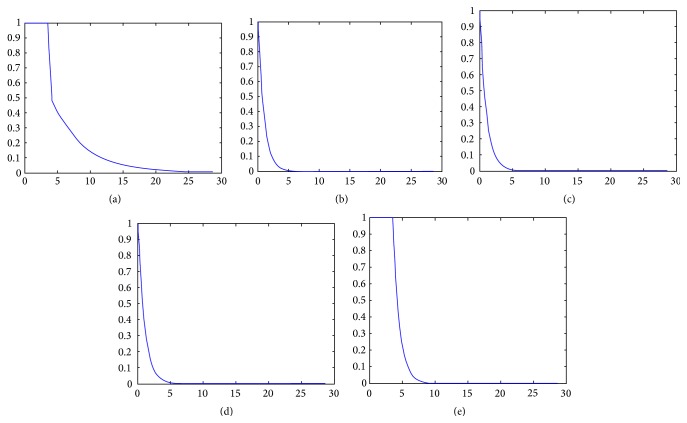


For extravasating contrast agents as used in MRI and CT, contrast agent molecules distribute across two spaces, that is, the intravascular space and the EES (Figure 3). Each space is defined by volume, rate, and transit time parameters. The relative intravascular plasma space is defined as the intravascular plasma volume relative divided by total tissue volume (*v*_*p*_). The intravascular flow rate (*F*) equals the blood perfusion rate per unit of volume of tissue and the mean capillary transit time (MTT_*c*_) is the ratio between the blood volume and the tissue blood perfusion rate. Similarly, the tissue interstitial volume (*v*_*e*_) is the sum of extravascular extracellular space (EES) volume contained in a volume of tissue. The two-way exchange rate to and from the EES is called the permeability-surface product, PS, and the MTT_*e*_ is the mean transit time for the EES. Additionally, an extraction fraction (*E*) describes the proportion of the contrast agent distributing to the EES. IRF is affected by the inflow of contrast (perfusion), two-way exchange of contrast between plasma and the EES, extraction fraction, and permeability. High-order perfusion models take into account such dynamics as completely as possible, although assumptions remain and additional variables do not necessarily yield more accurate results. The lower order models assume some parameters or dynamics to be negligible compared to others, thus simplifying the model. In Figure 4 [Fig fig5] each modeling approach is illustrated, with the formulation presented in Table 1.

## 5. Models Based on Axially Distributed Parameters 

### 5.1. Distributed Parameter Model

This model takes into account the most detailed aspects of contrast dynamics at the tissue level. It assumes contrast concentration within the intravascular space and EES to be varying temporally and axially along the longitudinal direction of the perfusion flow (Figure 4(a)). As such, the model is able to estimate every volume, rate, and time parameter specified in the intravascular and the interstitial space, as well as the extraction fraction. The distributed parameter model has been applied to estimate MRI stress/rest myocardial perfusion in healthy volunteers [37].

### 5.2. Tissue Homogeneity Model

This model by Johnson and Wilson assumes that the contrast concentration only varies longitudinally in the intravascular space and not in the EES (Figure 4(b)) [38]. With this assumption, the model loses the ability to estimate the time parameter of the EES (MTT_*e*_) but can still estimate the other intravascular and EES parameters. These two axially distributed models require special numerical treatments for model fitting (i.e., multiple or Laplace-domain fitting) due to their complexity [39, 40].

### 5.3. Adiabatic Approximation of Tissue Homogeneity Model

Developed by Lawrence and Lee, this model further simplifies the tissue homogeneity model by assuming that the contrast exchange between the intravascular space and the EES only takes place in the venous outlet [41]. Therefore, the rate of concentration change in the EES is much slower than in the intravascular space (Figure 4(c)). Adiabatic model fitting can be performed as a standard time-domain deconvolution with IRF, as specified in Table 1: height and length of the plateau correspond to perfusion flow and capillary mean transit time (MTT_*c*_), respectively, while the decay rate of the monoexponential function represents the venous clearance. This model was first proposed in brain studies but has been used in oncological and cardiac studies afterwards [42–46].

### 5.4. Implementation Issues

The main limitations for axially distributed models are (1) the need of a fast acquisition rate to support MTT_*c*_ estimation and (2) the more complicated and noise-sensitive fitting methods. Faster perfusion produces shorter MTT_*c*_, requiring more compact contrast bolus to accurately capture the MTT_*c*_ from the contrast dynamics.

## 6. Models Based on Compartments

The main difference between the compartmental and axially distributed model lies in the assumption that intravascular and EES contrast agent concentrations only vary with time, and not axially (Figures 4(d) and 4(e)). Because the axial contrast concentration gradient is considered negligible, transit time cannot be estimated, limiting the modeling results to the volume and rate parameters.

### 6.1. 2-Compartmental and 1-Compartmental Model

The typical IRF of a 2-compartment model takes the shape of a biexponential function, without an initial plateau for the capillary inflow phase due to the absence of transit time estimation. The faster-decaying exponential refers to the transfer towards the EES while the slower exponential refers to the transfer from the EES. On the other hand, the IRF of a 1-compartment model takes the shape of a monoexponential function. The maximum magnitude of the IRF corresponds to *K*_trans_, a compound tissue transfer constant formulated by multiplying perfusion (*F*) by the contrast extraction fraction (*E*) [47]. Three main 1-compartmental models are distinguished.

### 6.2. Toft's Models

The basic Toft's model refers specifically to immediate and complete tracer extraction fraction (*E* = 1) and negligible *v*_*p*_ compared to *v*_*e*_, such that *K*_trans_ represents perfusion flow [47]. Since *v*_*p*_ is not negligible in the myocardium, one study applied an Extended Toft's model where *v*_*p*_ is added to the original IRF [47, 48]. However, it has been argued that, under the Extended Toft's model, *K*_trans_ is closer to the EES exchange rate than to the perfusion flow [49].

### 6.3. Patlak Model

The Patlak model includes only data portions from the early phase of contrast arrival, when the contrast agent has not significantly filled the EES yet. Here, EES contrast concentration is not adequate to cause diffusion of contrast molecules back to the intravascular space. Under this assumption, *v*_*e*_ and *v*_*p*_ can be considered a single compartment with a single transfer rate (*K*_trans_) [50]. The temporal growth in the EES contrast concentration will be linear to the rate of contrast transfer to the EES (*K*_trans_) and the AIF contrast concentration. Therefore, *K*_trans_ of the Patlak model can be reconstructed from the slope of a correlation map between tissue contrast concentration and the area under the curve of the AIF. However, care should be taken to make sure that only appropriate data portions are used. The Patlak model has been used in human MRI studies of myocardial perfusion, with an acquisition rate matching every heartbeat, as well as in animals with the acquisition rate matching every other heartbeat [30, 48, 51].

### 6.4. Maximum Slope Method

The maximum slope method is derived from exactly the same assumptions as the Patlak model, therefore requiring the same portion of data points and suffering the same concerns. The Patlak-equivalent *K*_trans_ is derived by normalizing the maximum slope of the tissue concentration to the maximum (peak) concentration of the AIF. The tissue maximal upslope is calculated by linear fitting while the AIF peak is derived from gamma-variate fitting. The method has been implemented in an older study based on electron-beam CT as well as in more recent myocardial perfusion studies with dual-source CT [21, 52–54].

### 6.5. Implementation Issues

The main critique on the 1-compartment model is that *K*_trans_ is a multiplication of extraction fraction (*E*) and perfusion instead of a sole perfusion (*F*) indicator. A limited number of MRI studies have shown a nonlinearly decreasing extraction suggesting a limited range of *K*_trans_ proportionality with perfusion if this parameter is to be derived from gadolinium [18, 28–30]. Correcting *K*_trans_ for the extraction fraction (*E*) improved the correlation between Patlak-derived *K*_trans_ and microsphere perfusion in an animal experiment [51].

## 7. Models Based on Indicator Dilution Theory

### 7.1. The Fermi Model

The Fermi model was initially developed for studies with a purely intravascular indicator. Assuming an axially varying contrast concentration in the intravascular space, it was observed that the IRF of an intravascular indicator resembled the shape of the Fermi function [19, 55]. The amplitude, width of initial plateau, and subsequent curve decay rate of the fitted Fermi function represent perfusion, capillary mean transit time, and venous clearance rate, respectively (Table 1). For extravasating tracers, the validity of the Fermi model holds as long as the tracer concentration in the EES is substantially lower than in the intravascular space (*c*_*e*_ ≪ *c*_*p*_), a condition assumed to be attainable in the first-pass of tracer circulation [36]. The Fermi model has been used in many MR human and animal studies [2, 36, 56–60] and it has been used in one CT porcine study [32].

### 7.2. Model-Independent Deconvolution

The previous perfusion models have been driven by specific physical assumptions on the distribution of contrast agent in the tissue, culminating into exact IRF formulation. A model-independent approach attempts to overcome these tissue-specific assumption problems by applying more generic mathematical constraints in the IRF calculation. With the central-volume principle applied in the indicator dilution theory, the initial magnitude of the IRF is then assumed as perfusion regardless of the shape of the IRF [19, 35]. Studies with high data quality have shown excellent agreement of model-independent deconvolution with true perfusion (simulation study) and microspheres (porcine study, *n* = 3), as well as with PET in healthy volunteers (*n* = 5) [35, 61].

### 7.3. Implementation Issues

Since the indicator dilution approach does not presume any separation between the intravascular and the EES contrast dynamics, its perfusion estimation is uncorrected for EES exchange. Therefore, the same concern as in 1-compartment models, that is, the consistency of perfusion representation over the physiological range of perfusion, also applies to indicator dilution theory models.

## 8. Influence of Different Acquisition Settings

In the previous paragraphs, the different perfusion models were discussed. Those models offer different degrees of perfusion evaluation. When more accurate quantification of perfusion is desired, consequently, more detailed information of contrast dynamics is required. This leads to more demanding acquisition settings (i.e., faster acquisition rate, higher contrast-to-noise ratio). As a result, more detailed models are more sensitive to noise, because a small change in the contrast dynamics will have more impact on the parameter estimations.

### 8.1. Key Acquisition Factors

Jerosch-Herold performed a thorough review on specific MRI requirements [19]. Minimal requirements of several general key acquisition/image quality parameters that influence the output of tracer kinetic models are listed here.

(1) A compact contrast bolus is needed to ensure that the contrast dynamics contains information as requested by the modeling. An increasingly dispersed bolus is known to cause increasing perfusion underestimation and variability, especially at higher flow rate [62]. As a rough guidance, the contrast bolus should be compact enough to accommodate a clear definition of the peak enhancement in the AIF as well as in the tissue (and even more compact in case of the use of the axially distributed model). A gadolinium injection rate of at least 3 mL/s, and optimally 4 mL/s, has been recommended for MRI myocardial perfusion assessment [18, 63]. More prominent bolus dispersion can be expected in CT due to the typically larger injected contrast volume.

(2) In order to estimate the flow rate and transit time parameters, a sufficiently fast acquisition rate (temporal resolution) is needed to capture the fastest change described by the model. When only the flow rate parameter is analyzed, the minimum scan interval is determined by the time-to-peak (TTP) of the AIF. When both rate and transit time parameters are concerned, the mean capillary transit time (MTT_*c*_) determines the minimum scan interval. In other tissues a considerable underestimation was found when the temporal resolution was reduced, with both CT and MRI [62, 64–68].

(3) In order to estimate volume parameters, the acquisition period should be at least within the order of the transit time parameter of the concerned volume, to ensure proper capture of the arrival and clearance of contrast agent.

(4) In-plane spatial resolution should be adequate to prevent partial volume effects, especially if voxel-wise tracer kinetic modeling is to be applied. In the data acquisition this means that voxels are best small and isotropic (cubic rather than rectangular, etc.). This is hard to realize in MRI where the in-plane spatial resolution is approximately 5 times lower than in CT (1.5 × 1.5 mm versus 0.3 × 0.3 mm) with slice thickness much larger than in-plane resolution. In postprocessing, the quantification resolution particularly worsens due to partial volume effects in CT where investigators have typically analyzed the CT perfusion in slices thicker than the native resolution.

(5) Signal-to-noise ratio (SNR) concerns the total variability in the contrast dynamics. Small variations in contrast dynamics may influence the precision of tracer kinetic modeling. The use of higher Tesla machines in MRI may provide better SNR without compromising spatiotemporal resolution, although inherent problems with RF homogeneity can adversely impact quantification. Implementation of higher tube current in CT can also improve SNR by reducing variability in contrast dynamics and the error in model fitting [19, 55]. A disadvantage of higher tube current is the increase in radiation dose. A decrease in tube voltage could increase SNR, because of the *K*-edge of iodine, which lies around 35 kV. If possible, a lower tube voltage would be beneficial for contrast scans and additionally lowers radiation dose [69].

## 9. Clinical Implication and Conclusion

Regarding the choice of model used, we suggest that one should use the simplest possible model that can explain the contrast dynamics. It is worth noting that the use of higher-order models will only be beneficial when the acquisition is optimized to capture the additional contrast dynamic details requested by the model. Considering the current imaging and contrast administration setup for MRI and CT myocardial perfusion imaging, the 1-compartmental and Fermi models seem to be the most technically applicable. Axially distributed models require an acquisition rate at the order of MTT_*c*_ and a sufficiently compact bolus to identify the capillary inflow phase. Balancing such demand with clinical requirements for spatial resolution and coverage could be problematic. Apart from optimal data quality, model-independent deconvolution also requires knowledge for selection of the regularization parameter, which may not be available in every imaging center.

Issues for clinical adoption go beyond the accuracy of the quantitative myocardial perfusion value itself. The assumptions made by each model are coupled with the theoretical pitfalls we have tried to identify in our appraisal.

The major complication with quantitative MR perfusion is in the limited linearity of contrast enhancement to contrast concentration, requiring lower dose of gadolinium, thus compromising the accuracy of visual analysis as well as the precision of perfusion estimation. CT perfusion on the other hand greatly simplifies quantification efforts by offering a linear relationship between contrast enhancement and concentration. However, current CT imaging setup suffers reduced image quality due to the shuttling mode acquisition and limited temporal resolution as well as acquisition period due to the radiation dose constraint; both reduce the precision and accuracy of perfusion estimation.

Limited studies have mentioned instability issues of higher-order models. More investigations are required [37, 70]. Reproducibility of perfusion values is highly related to the imaging quality, where specific issues such as acquisition/reconstruction artifacts need to be taken care of in both modalities before implementing the model.

An important issue for the clinical setting would be to establish the expected physiological variability across different subjects, so that the usability of quantitative myocardial perfusion in diagnostic or sequential observation setting can be verified. Quantitative PET studies for instance have shown considerable heterogeneity in myocardial perfusion of healthy volunteers, related to factors such as age, gender, rate-pressure product, and other hemodynamic factors [71, 72]. Furthermore, in the presence of COPD and hypertension, the value of myocardial perfusion reserve has been shown to be impaired without regional myocardial ischemia [73–75]. The spectrum of physiological variability in myocardial perfusion should also be investigated with MRI and CT if myocardial perfusion quantification is to be adopted in clinical practice.

Studies investigating the performance of quantitative MRI and CT myocardial perfusion imaging in detecting CAD have been conducted with different reference standards, that is, stenosis diameter, derived from either quantitative CT or invasive coronary angiography, fractional flow reserve, or even visual analysis of SPECT myocardial perfusion. None of these reference parameters actually capture the same functional phenomenon as myocardial perfusion. The anatomical aspect of a stenosis does not describe its functional relevance, while fractional flow reserve, even though being a functional parameter, indicates the hemodynamics of the focal coronary lesion rather than its systemic effect on myocardial microcirculation. The quantitative relationship between myocardial perfusion and the above parameters, therefore, can be expected to be affected by broader physiological variability, which may be better captured by quantitative analysis than by visual assessment. However, the superiority of quantitative myocardial perfusion over mere visual analysis for diagnosis of hemodynamically significant CAD still needs to be proven.

## Figures and Tables

**Table 1:** Tracer kinetic model formulation.

Model	Output parameters	Impulse response function (IRF)
Distributed parameter	*F*, PS, MTT_*c*_, MTT_*e*_, *v*_*p*_, *v*_*e*_	Not available in time domain
Tissue homogeneity	*F*, *E*, MTT_*c*_, *v*_*p*_, *v*_*e*_	

Adiabatic approximation of tissue homogeneity	*F*, E, MTT_*c*_, *v*_*p*_, *v*_*e*_ (assuming *v*_*p*_ ≪ *v*_*e*_)	See Figure 5(a) $IRF\left(t\right)=\left\{\begin{array}{cc} F, & 0<t\leq \frac{F}{v_{p}}\\ EF\;{exp}^{-\left(EF/v_{e}\right)\left(t\right)}, & t>\frac{F}{v_{p}} \end{array}\right.$

2-compartment	*F*, PS, *v*_*p*_, *v*_*e*_	See Figure 5(b) IRF(*t*) = *F* exp^−(*F*/*v*_*p*_)(*t*)^ + PS exp^−(PS/*v*_*e*_ )(*t*)^

1-compartment (Extended Toft's)	*K*_trans_, *v*_*p*_, *v*_*e*_	See Figure 5(c) IRF(*t*) = *K*_trans_ exp^−(*K*_trans_/*v*_*e*_)(*t*)^ + *v*_*p*_∂(*t*)

1-compartment (Toft's)	*K*_trans_, *v*_*e*_ (assuming *v*_*p*_ ≪ *v*_*e*_)	See Figure 5(d) IRF(*t*) = *K*_trans_ exp^−(*K*_trans_/*v*_*e*_)(*t*)^

Fermi	*F*, MTT_*c*_, *k* (in extravasating contrast agent, only *F* is of physiological value)	See Figure 5(e) $IRF\left(t\right)=\frac{F}{{exp}^{k\left(t-{MTT}_{c}\right)}+1}$

Model-independent deconvolution	*F* (estimated as initial IRF magnitude)	No specific formulation

*F*: perfusion rate.

PS: extracellular extravascular space (EES) exchange rate.

MTT_*c*_: capillary mean transit time.

MTT_*e*_: EES mean transit time.

*v*_*p*_: EES volume fraction.

*v*_*e*_: intravascular plasma volume fraction.

*K*_trans_: compound transfer constant (perfusion and EES exchange).

*k*: venous clearance rate for intravascular contrast agent.
